# Breed group differences in the unsolvable problem task: herding dogs prefer their owner, while solitary hunting dogs seek stranger proximity

**DOI:** 10.1007/s10071-021-01582-5

**Published:** 2021-11-18

**Authors:** Enya Van Poucke, Amanda Höglin, Per Jensen, Lina S. V. Roth

**Affiliations:** Linköping University, IFM Biology, 581 83 Linköping, Sweden

**Keywords:** Dog, Dog–human relationship, Contact-seeking behaviour, Unsolvable problem task

## Abstract

The communicating skills of dogs are well documented and especially their contact-seeking behaviours towards humans. The aim of this study was to use the unsolvable problem paradigm to investigate differences between breed groups in their contact-seeking behaviours towards their owner and a stranger. Twenty-four dogs of ancient breeds, 58 herding dogs, and 17 solitary hunting dogs were included in the study, and their behaviour when presented with an unsolvable problem task (UPT) was recorded for 3 min. All breed groups interacted with the test apparatus the same amount of time, but the herding dogs showed a longer gaze duration towards their owner compared to the other groups and they also preferred to interact with their owner instead of a stranger. Interestingly, the solitary hunting dogs were more in stranger proximity than the other groups, and they also showed a preference to make contact with a stranger instead of their owner. Hence, we found differences in contact-seeking behaviours, reflecting the dog–human relationship, between breed groups that might not only be related to their genetic similarity to wolves, but also due to the specific breeding history of the dogs.

**Supplementary Information:**

The online version contains supplementary material available at 10.1007/s10071-021-01582-5.

## Introduction

The domestic dog is well known for its abilities to communicate with humans (Miklósi et al. 2000) and this ability is already present at an early age (Passalacqua et al. 2011). One commonly used method to trigger dogs’ communication with humans is to present the dog with an unsolvable problem, and in a pioneering study Miklósi et al. (2003) compared the contact-seeking behaviours of dogs to those of similarly socialised wolves. They found that dogs made quicker and longer eye contact than their ancestors, the wolves, and since then, various unsolvable problem tasks (UPT) have been used to study the effects of breed, age, sex, etc., on the contact-seeking behaviours of dogs (see reviews by Cavalli et al. 2018; Mendes et al. 2021).

Today, there are over 400 dog breeds officially recognised by the Fédération Cynologique Internationale, and these are further divided into breed groups depending on their phenotypical traits, such as behavioural skills, appearance, but also according to their genetic origin. Genetically closest to the ancestral wolf are the ancient breeds (Parker et al. 2017), such as Shiba Inu and basenji, which are not specifically bred for human cooperation. On the other hand, we have the herding dogs, such as border collies, that are heavily selected for human cooperation. There are also breeds that are primarily selected for their sensory skills and natural behaviour, such as hunting dogs. In Scandinavia there is even a special type of hunting dog that is released into the forest during the hunting season to work on its own, separated from the owner. These solitary hunting dogs are not specifically selected for human cooperation and contact-seeking behaviour, but instead for their skills of searching the terrain and tracking down prey animals using mainly their olfactory sense. These dog breeds can belong to different genetically divided breed groups, and examples of solitary hunting breeds are Swedish and Norwegian elkhounds, dachshunds, and hunting terriers.

Hence, hunting breeds can be diverse and include both solitary hunting breeds and breeds that work in close proximity to humans, such as the retrievers. Passalacqua et al. (2011) found that their retrieving and herding breeds gazed for a longer duration compared to ancient dog breeds. In addition, Maglieri et al. (2019) found that the genetic closeness to wolves reduced contact-seeking behaviours, and that their group of retrievers gazed the most towards humans. In this study, we investigated contact-seeking behaviour in actively used solitary hunting breeds, bred for hunting independently of humans, and compared them to herding dogs selected for human cooperation, and also to ancient breeds, genetically closer to the wolf.

Recently, we have shown that herding dogs synchronize with their owners in long-term stress levels (Sundman et al. 2019), while solitary hunting dogs and ancient dog breeds do not (Höglin et al. 2021). The aim of this paper was to investigate the behavioural differences between these three breed groups, using the same dogs, focusing on the contact-seeking behaviour towards both the owner and a stranger using an unsolvable problem task (UPT). We hypothesized that herding dogs would show more contact-seeking behaviours towards the owner compared to the other breed groups, but also that the preference for the owner could be less obvious in the breeds that are not specifically selected for human cooperation.

## Method

### Subject information

Dogs and their owners were recruited through social media and personal contacts, and consisted of 24 dogs belonging to ancient dogs (15 females and 9 males) with a mean age of 4.83 years ± 0.60 SE, 17 solitary hunting dogs (14 females and 3 males) with a mean age of 5.06 years ± 0.84 SE, and 58 herding dogs (23 females and 35 males) with a mean age of 4.7 years ± 0.38 SE. The herding dogs could also be divided into 32 competing and 26 companion (non-competing) dogs, where competing dyads reported that they actively trained and competed in either agility, obedience, or both disciplines. All dogs in this study lived indoors as pet dogs, even though the solitary hunting dogs were also actively used for hunting purposes. For more information about the dogs and their specific breeds see Supplementary 1.

### The unsolvable problem task

**Fig. 1:**
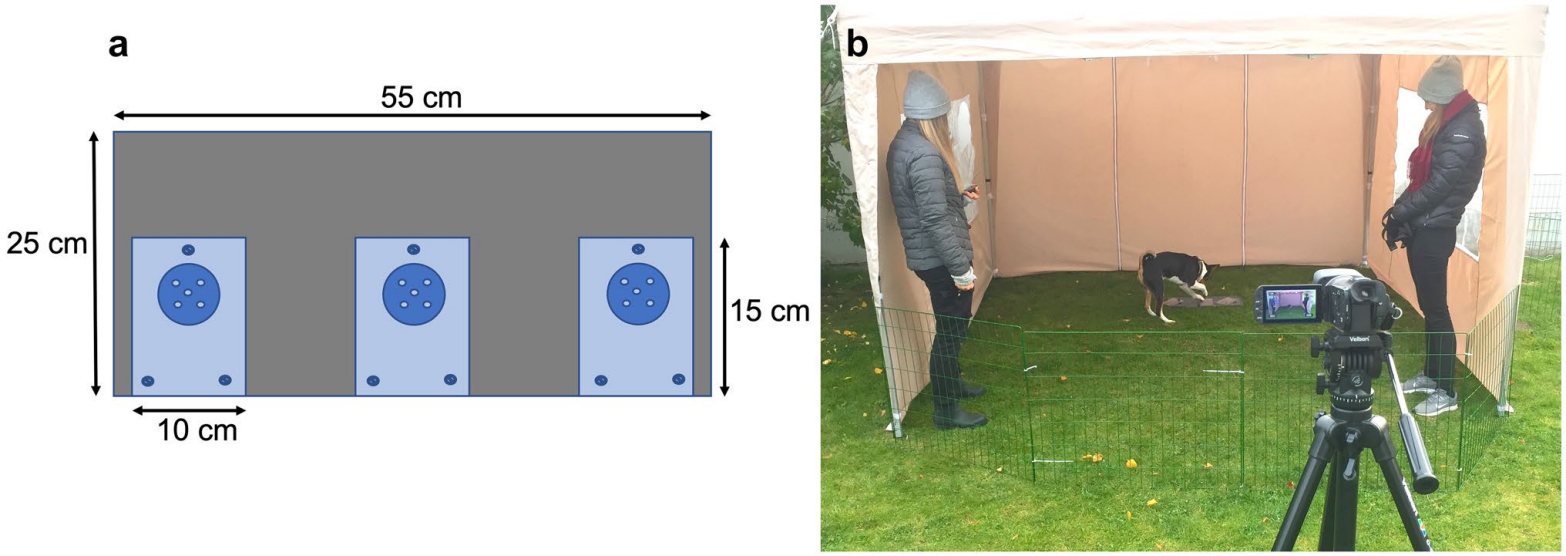
**a** Test apparatus consisted of two outer solvable compartments and one middle unsolvable compartment. The apparatus was placed **b** in the middle back of the test arena with the owner and experimenter standing in each front corner of the marquee

The behavioural experiment took place outdoors at Linköping University, southeast of Sweden, during September–October in 2018 and 2019. The UPT, consisting of both two solvable and one unsolvable task, has previously been described in detail by (Persson et al. 2015; Sundman et al. 2018). In short, the apparatus consists of three compartments, where the outer two lids are possible to slide to the side, making the treat accessible to the dog (Fig. 1a). The lid covering the middle compartment is fastened and hence, unsolvable. Before being presented with the UPT the dog’s motivation was tested by the female test leader. This was done with a separate compartment without lid, wherefrom the dog was allowed to eat three treats. If the dog succeeded, the owner and an unfamiliar female experimenter walked into their positions within the marquee that was novel to the dog. The experimenter placed herself in the front left corner, and the owner and the dog in the front right corner (Fig. 1b). Then the test leader positioned the UPT apparatus in the middle of the back side of the marquee, and tapped with her finger on the apparatus to obtain the dog’s attention. When the dog looked towards the UPT apparatus it was released and the test leader left the marquee and the surrounding test area. During the following 3 min the experimenter and the owner were motionless, ignored the dog, and faced the UPT apparatus. However, if the dog did not manage to open any of the solvable tasks within the first minute, both experimenter and owner would simultaneously walk up and each open one lid halfway, and then return to their initial positions. The dog’s behaviour during 3 min was video recorded (Canon Legria), and later continuously recorded using the software Observer XT (Noldus) with a predetermined ethogram (Table 1). Note that the hair samples used to assess long-term stress levels in Sundman et al. (2019) and Höglin et al. (2021) were obtained after the behavioural part of the study (and could, therefore, not have affected the behavioural test).

**Table 1:** Ethogram used in the behaviour analysis of the problem-solving test, adapted from Persson et al. (2015)

Behaviour	Definition
Eye contact—owner	The dog’s head directed towards the owner
Eye contact—experimenter	The dog’s head directed towards the experimenter
Proximity—owner	The dog’s head within its own body length of the owner
Proximity—experimenter	The dog’s head within its own body length of the experimenter
Proximity—test apparatus	The dog’s head within its own body length of the test apparatus
Physical contact—owner	The dog in physical contact with the owner
Physical contact—experimenter	The dog in physical contact with the experimenter
Physical contact—test apparatus	The dog in physical contact with the test apparatus

### Data analysis

The behavioural data were not normally distributed, and therefore, non-parametrical tests were used, using the software IBM SPSS Statistics (version 27). Inter-rater reliability between two observers was tested using Spearman’s correlation for the human-related behaviours in 10% of the dogs, which revealed high reliability (*rs* = 0.89, *p* = 0.001).

When comparing breed groups independent Kruskal–Wallis tests were used and pairwise comparisons were adjusted by the Bonferroni correction for multiple tests. To compare the breed groups’ behaviour towards owner and experimenter Wilcoxon Signed-Rank tests were used.

The behavioural interaction with the test apparatus was correlated with eye-contact related behaviours (including latencies for eye contact) using Spearman’s Correlations. Ages of the dog were also correlated with behaviours using Spearman’s Correlations. Mean and SE is reported in the results.

## Results

### Breed group differences

Since there were no significant differences in recorded behaviour between competing and non-competing herding dogs, these dogs were considered as one single group. In addition, there were no significant sex differences within breed groups, so females and males were pooled together (see Supplementary 2 for these non-significant results).

**Fig. 2:**
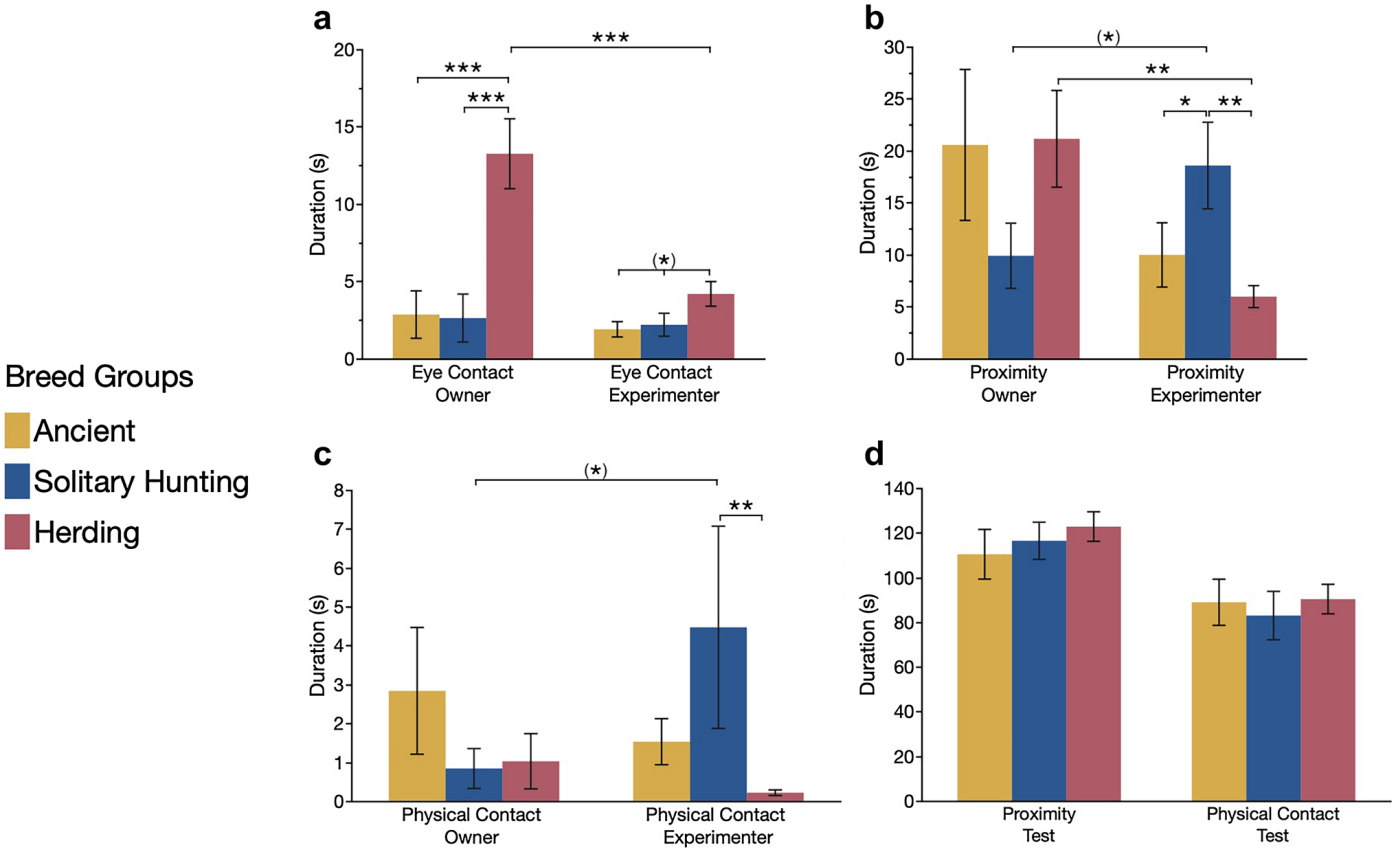
Mean duration (s ± 1SE) of **a** eye contact-seeking behaviour, **b** proximity, **c** physical contact, towards the owner and the stranger, and **d** interaction with the test apparatus, for ancient breed group, solitary hunting dogs and herding dogs *** *p* < 0.001, ** *p* < 0.01, * *p* < 0.05, (*) *p* < 0.1

Eye contact-seeking behaviour towards the owner differed significantly between the three breed groups (χ^2^ = 24.80, *p* < 0.001; Fig. 2a), where herding dogs showed longer duration of eye contact than both solitary hunting breeds (*p* < 0.001) and ancient dog breeds (*p* < 0.001). There was also a tendency for eye contact-seeking behaviour towards the unfamiliar experimenter to differ between breed groups (χ^2^ = 5.32, *p* = 0.070; Fig. 2a).

Proximity behaviour towards the owner did not differ between breed groups (χ^2^ = 0.39, *p* = 0.824; Fig. 2b). However, proximity towards the experimenter differed significantly (χ^2^ = 11.63, *p* = 0.003), where the solitary hunting dog breeds revealed longer duration of experimenter proximity than both ancient (*p* = 0.034) and herding breeds (*p* = 0.002; Fig. 2b).

Similarly, there was no difference between breed groups in duration of physical contact with the owner (χ^2^ = 1.71, *p* = 0.425; Fig. 2c), while we found significant differences for experimenter physical contact (χ^2^ = 9.55, *p* = 0.008). Again, the solitary hunting breed group revealed longer duration of physical contact with the experimenter than herding dogs (*p* = 0.008), but there was no significant difference compared to ancient dogs (*p* = 0.42; Fig. 2c).

Age did not correlate with any of the behaviours (*p* > 0.1; See supplementary 2).

### Owner and stranger preferences

Comparing the behaviour towards owner and the unfamiliar experimenter revealed that herding dogs showed significantly more eye contact-seeking behaviour with the owner compared to the experimenter (*N* = 58, *z* = −3.67, *p* < 0.001; Fig. 2a). In addition, herding dogs were also significantly more in owner proximity than experimenter proximity (*N* = 58, *z* = −3.02, *p* = 0.003; Fig. 2b). On the contrary, solitary hunting dogs tended to show both more proximity (*N* = 17, *z* = −1.71, *p* = 0.088) and physical contact (*N* = 17, *z* = −1.96, *p* = 0.050) behaviours towards the experimenter compared to the owner.

### Test apparatus-related behaviours

There was no difference between breed groups in test apparatus proximity (χ^2^ = 1.17, *p* = 0.56), and similarly, no difference for physical contact with the test apparatus (χ^2^ = 0.46, *p* = 0.80; Fig. 2d).

The dog’s proximity to the test apparatus correlated significantly with the latency to make eye contact with both the owner (*N* = 99, *rs* = 0.26, *p* = 0.009) and experimenter (*N* = 99, *rs* = 0.45, *p* < 0.001). Similarly physical contact with the test apparatus correlated significantly with latency to make eye contact with the owner (*N* = 99, *rs* = 0.29, *p* = 0.004) and experimenter (*N* = 99, *rs* = 0.46, *p* < 0.001).

Age did not correlate with any of the behaviours (*p* > 0.1; See Supplementary 2).

## Discussion

The aim of this study was to investigate three different breed groups with regard to their human contact-seeking behaviour. Using the unsolvable problem task, we found that herding dogs gazed longer towards the owner compared to both solitary hunting breeds and ancient breeds. In addition, while herding dogs preferred their owner, the solitary hunting dogs showed most contact-seeking behaviours towards the unfamiliar experimenter.

In our study, herding dogs showed a much longer eye contact duration during UPT compared to the other breed groups, which is in line with Passalacqua et al. (2011). However, we found no difference between the ancient breed group and the solitary hunting breeds, indicating that not only relatedness to the wolf affects the eye contact-seeking behaviour as suggested by Konno et al. (2016), but also selection and function of the dog breed as suggested by Passalacqua et al. (2011). In Passalacqua et al. (2011) both herding dogs and retrieving breeds are grouped together as a hunting/herding group, which might give the impression that all hunting breeds are selected for human cooperation and show much human contact. Our solitary hunting dogs gazed only short durations towards humans during the UPT, which is also different from the hounds in Konno et al. (2016) that found similar gazing durations for all breed groups except for the ancient breeds that gazed the least. In both studies the experimenter was training the dogs in solvable tasks before the task was made unsolvable. This might increase some dogs’ contact-seeking behaviour due to the association between treats and the experimenter. In our UPT, the experimenter was not associated with either treats, the motivation test plate, or the actual test apparatus and was, therefore, a neutral stranger to the dog.

In this study, both the owner and an unfamiliar experimenter were present during the UPT, allowing the dog to choose whom to seek contact with. Since we analysed the contact-seeking behaviour towards the humans separately it was possible to study whether the dog revealed a preference. As hypothesized, we found differences in human preference between the breed groups. While the herding dogs gazed longer towards the owner and were more in proximity to the owner compared to the unfamiliar experimenter, the solitary hunting dogs preferred to be in experimenter proximity. In previous UPT studies, there is usually a preference for the owner or no obvious preference (see review Cavalli et al 2018, but note Maglieri et al. 2019). Note, however, that the preference in the solitary hunting dogs in this study refers to the proximity and physical contact to the experimenter, and not eye contact-seeking behaviour. Still, the results might indicate that solitary hunting dogs are curious towards strangers which could be a result from experience, hunting in large teams, or it could be related to personality or breed traits. Since this study is part of a larger study, the personality of these dogs has been investigated previously (Höglin et al. 2021). However, the only personality trait, where the solitary hunting dogs differed significantly from the other groups was *Activity/Excitability*, where herding dogs revealed the highest scores. Another possible reason for the experimenter preference in the solitary hunting dogs could be differences in the human–animal relationship as suggested by Cavalli et al. (2018) and Mendes et al. (2021). Indeed, as reported earlier in Höglin et al. (2021), the owner-reported relationship scores (assessed by MDORS) for both the subscale *Dog*–*Owner Interaction* and *Perceived Emotional Closeness* were lower for the solitary hunting dogs compared to both ancient dog breeds and herding dogs. In addition, the score for the subscale *Perceived Cost* was high for solitary hunting dogs compared to the other breed groups. Hence, in line with Topál et al. (1997), where the type of relationship was linked to the dog’s behaviour, this weaker relationship might be related to why the solitary hunting dogs seek more contact with an unfamiliar experimenter instead of their owner. However, note that ancient dog breeds and herding dogs were similar in their relationship scores but still differed in their contact-seeking behaviour in the UPT in this study. Hence, future studies should investigate this human preference further to disentangle the effect of breed group and the human–dog relationship.

In addition to breed group, the dog’s training experiences is suggested to influence the gazing behaviour, and Marshall-Pescini et al. (2016) found that dogs that are more trained gaze less towards humans during a problem-solving task compared to non-trained dogs. In our study, we did not find any differences between the two lifestyles within the herding dogs, i.e., actively competing dogs (in agility or obedience) and dogs kept as pet dogs. This could suggest limited effect of training in the herding group, but the dog’s training experience could still be important to consider when investigating contact-seeking behaviour in other breeds. Topál et al. (1997) found untrained dogs to play more with strangers, which might add to the explanation for the solitary hunting dogs’ behaviour towards the unfamiliar experimenter in our study. However, since training activities were not assessed for the ancient and solitary hunting breed group we will not speculate further on this point.

Also, one limitation of this study is that there were relatively more males in the herding group compared to the other breed groups. Even though we did not find any sex differences and, therefore, pooled the data, there might be a skewness that affects the results. However, in studies testing for possible sex differences in UPT, sex has been suggested to have little effect on the gazing behaviour (Konno et al. 2016; Passalacqua et al. 2011; Persson et al. 2015; Sommese et al. 2019; Topál et al. 1997) but note that female laboratory beagles show higher proximity to humans than male beagles (Persson et al. 2015).

Genetics and relatedness to the wolf is, as earlier mentioned, suggested to play a key role in eye contact-seeking behaviour of dogs (Konno et al. 2016; Maglieri et al. 2019; Sommese et al. 2019), where breeds more closely related to wolves gaze the least towards humans during an UPT. In our study, both ancient dog breeds and solitary hunting breeds showed little gazing behaviour towards humans. However, since our solitary hunting dogs belonged to different breed groups it is difficult to fully untangle selective breeding for solitary hunting behaviour and relatedness to the wolf in this study.

The persistence in the UPT has been associated to the latency to seek eye contact with humans and has, therefore, been raised as an issue when comparing animals in their contact-seeking behaviour (Mendes et al. 2021). Indeed, we did find correlations between persistence and latency in seeking eye contact, similar to Marshall-Pescini et al. (2017). However, we found no difference in persistence in the UPT between groups, since all breed groups interacted with the test apparatus for a similar amount of time. Therefore, the differences we found in contact-seeking behaviour between groups in this study cannot be explained by the dogs’ persistence and motivation for the task.

In conclusion, all dogs showed similar interest in the UPT and while the herding dogs gazed longer at the owner, the solitary hunting dogs revealed a preference for the unfamiliar experimenter which might be linked to both breed selection and differences in the dog–human relationship.

## Supplementary Information

Below is the link to the electronic supplementary material.

Supplementary file1 (XLSX 20 KB)

Supplementary file2 (XLSX 13 KB)

## Data Availability

All data generated or analysed during this study are included in this published article, and its supplementary information files.
